# Effect of dietary Ca:P ratio on ionized calcium and calcium homeostasis in cats with early-stage chronic kidney disease

**DOI:** 10.1371/journal.pone.0350414

**Published:** 2026-05-29

**Authors:** Jean A. Hall, Leslie B. Hancock, Elizabeth M. Morris

**Affiliations:** 1 Department of Biomedical Sciences, Carlson College of Veterinary Medicine, Oregon State University, Corvallis, Oregon, United States of America; 2 Pet Nutrition Center, Hill’s Pet Nutrition, Incorporated, Topeka, Kansas, United States of America; University of Life Sciences in Lublin, POLAND

## Abstract

The aim of the study was to compare two renal foods with different Ca:P ratios on manifestation of hypercalcemia and regulation of calcium homeostasis in cats with chronic kidney disease (CKD). Nine cats (11.0 ± 2.0 y) with naturally-occurring IRIS Stage I or II CKD were fed a senior wellness food for 28-days, then randomized into two groups and fed either a food providing 1.8 g/Mcal Ca, 1.3 g/Mcal P, and Ca:P ratio of 1.4:1 (MOD-Ca:P), or a food providing 2.4 g/Mcal Ca, 1.3 g/Mcal P, and Ca:P ratio of 1.8:1 (HIGH-Ca:P) for 56 days. After a 28-day washout period, cats were crossed over to the other test food. Blood and urine samples were collected at the end of the prefeed/washout and on days 28 and 56 of each study period. Data were analyzed using a linear mixed model with fixed effects of Diet, Day, Period and associated interactions. At baseline, mean ionized calcium (iCa; 1.26 ± 0.03 mmol/L) was within the normal reference interval (1.10–1.40 mmol/L). Serum total Ca, phosphorus, calcitriol, and fractional excretion of calcium were not affected by diet and diet by day interaction (*P* > 0.050). There was a trend for increased fibroblast growth factor 23 (*P* = 0.058) and serum iCa (*P* = 0.067) in cats fed HIGH-Ca:P compared with MOD-Ca:P food, although iCa remained within the normal reference interval. Parathyroid hormone was reduced when cats were fed HIGH-Ca:P compared with cats fed MOD-Ca:P (*P* = 0.020), although concentrations were within the normal reference interval throughout the study. Fractional excretion of phosphorus was reduced when cats were fed HIGH-Ca:P compared with MOD-Ca:P (*P* = 0.050). Serum Cr and SDMA were higher on days 28 and 56 compared with baseline (*P* < 0.002). Higher dietary Ca and a higher Ca:P ratio may not be the sole drivers of hypercalcemia in cats with progressive CKD. However, the observed alterations in calcium homeostatic mechanisms warrant further research to identify strategies for mitigating hypercalcemia risk.

## Introduction

Feeding a therapeutic renal food with restricted phosphate in cats with early chronic kidney disease (CKD; International Renal Interest Society, IRIS, stage 2) is recommended to prevent hyperphosphatemia [1,2]. A decrease in glomerular filtration rate (GFR) results in phosphate retention. Decreased renal function results in decreased activity of the 1α-hydroxylase enzyme, and decreased production of calcitriol. Increased plasma phosphate concentrations stimulate fibroblast growth factor 23 (FGF-23) secretion [3]. In early CKD, increased FGF-23 increases the fractional excretion of phosphate and also inhibits calcitriol production in the kidney. In late-stage CKD, fractional excretion of phosphate cannot be further increased due to loss of nephron mass/function. As a result, hyperphosphatemia develops. Hyperphosphatemia, along with decreased calcitriol and decreased ionized calcium (iCa), leads to increased parathyroid hormone (PTH) secretion and secondary renal hyperparathyroidism, the latter characterized by demineralization of bone and soft tissue mineralization [4–6]. Hyperphosphatemia also predicts progression of kidney disease and shortens renal survival time [7–11].

Dietary phosphate restriction, may also contribute to the development of hypercalcemia [12,13]. Increased total serum calcium (tCa) and iCa are common in cats with azotemic CKD and are associated with progressive loss of renal function [12,14–16]. Up to 30% of cats with CKD developed hypercalcemia in one study [16]. Several retrospective studies have shown that cats in early stage CKD develop hypercalcemia after long-term feeding of highly phosphate-restricted food, whereas feeding moderately phosphate-restricted food improved calcium and phosphate balance [13,15]. In a previous study [17], we showed that feeding a highly phosphate-restricted food with a high Ca:P ratio (2.3 g/Mcal calcium; 1.1 g/Mcal phosphorus; Ca:P ratio, 2.0:1) to cats with early stage CKD was likely to cause increased serum iCa, tCa, and FGF-23 concentrations, and result in higher fractional excretion of calcium in the urine, whereas cats fed a moderately phosphate-restricted food (1.8 g/Mcal calcium; 1.5 g/Mcal phosphorus; Ca:P ratio, 1.2:1) maintained normal iCa and tCa, with no evidence of hyperphosphatemia, although renal secondary hyperparathyroidism persisted in some cats.

Phosphate-restricted foods may cause hypercalcemia because phosphate restriction increases the Ca:P ratio. Therefore, the potential cause(s) for hypercalcemia in cats fed therapeutic renal foods must take into consideration the absolute Ca content and the Ca:P ratio. The objective of this study was to evaluate the impact of feeding renal foods with different Ca:P ratio, but similar P content, on serum iCa and the associated hormonal and regulatory mechanisms for maintaining calcium homeostasis in cats with CKD. The underlying hypothesis was that food with a higher Ca:P ratio would negatively impact serum iCa and its homeostatic mechanisms compared with a food having a lower Ca:P ratio.

## Methods

### Animals and study design

All study protocols were reviewed and approved by the Institutional Animal Care and Use Committee, Hill’s Pet Nutrition, Inc., Topeka, KS (Permit Number: CP1104). Each cat had an annual physical examination, complete blood count (CBC), serum biochemical analyses, urinalysis, and urine culture if indicated by the urinalysis results. Cats were housed individually and allowed exercise in indoor runs. Cats had access to natural light that varied with seasonal changes. All study animals were allowed normal socialization and enrichment activities and the design of the study did not interfere with the animals’ normal daily routine. All cats were provided with regular opportunities to exercise, with access to toys. All cats were owned by the commercial funders of this research or their affiliates, who gave permission for them to be included in this study.

Eleven cats were chosen from the colony of domestic short-hair cats. Criteria for inclusion were age > 1 year with a body condition score >1 using a 1–5 scale, where 1 is extremely underweight and 5 is obese, and blood and/or urine parameters that were consistent with IRIS stage 1 or 2 CKD [18]. Cats were excluded from the study if they were considered healthy, or if they had been diagnosed with diseases other than CKD, including, but not limited to pancreatitis, hyperthyroidism, or cancer.

Chosen cats were 11.0 ± 2.0 years of age, 4 neutered males and 7 ovariohysterectomized females, with an initial body weight (BW) of 4.88 ± 0.86 kg. Cats enrolled in the study could subsequently be removed from the study if they experienced loss of >10% BW despite intervention, if they stopped eating or ate < 50% of food ration for 3 consecutive days, if they were diagnosed with any secondary systemic disease as described in exclusion criteria, or if an adverse event occurred that required removal from the study at the discretion of the attending veterinarian.

The trial was a randomized crossover study design with two 56-day treatment periods and a 28-day washout period between treatment periods. After a 28-day prefeed in which cats were fed a senior wellness cat food, cats were stratified by BW, sex, age, serum symmetric dimethylarginine (SDMA), serum creatinine (Cr), and IRIS stage, and then randomized into one of two experimental dietary treatment groups. The ingredient profiles of prefeed and test foods are shown in Table 1, and the analyzed nutrient composition of each food on both a dry matter and caloric basis are shown in Table 2. Nutrient composition was determined by a commercial laboratory (Eurofins Scientific, Inc., Des Moines, IA). The first test food provided 1.8 g/Mcal Ca, 1.3 g/Mcal P, and a Ca:P ratio of 1.4:1 (**MOD-Ca:P**; *n* = 5 cats Period 1), and the other test food provided 2.4 g/Mcal Ca, 1.3 g/Mcal P, and a Ca:P ratio of 1.8:1 (**HIGH-Ca:P**; *n* = 6 cats Period 1). Calcium sulfate and calcium carbonate were added at the expense of whole red wheat to increase calcium in the HIGH-Ca:P food.

**Table 1:** Major ingredients of test foods.

	Study Foods	
Ingredient, %^a^	HIGH-Ca:P^b^	MOD-Ca:P^c^
Brown Rice	17.5	17.5
Corn Protein Meal	17.5	17.5
Chicken	14.9	14.9
Chicken Fat	11.2	11.2
Pea Protein	4.2	4.2
Brewers Rice	9.0	9.0
Whole Grain Oats	0.4	0.4
Whole Grain Wheat	5.2	6.0
Chicken Liver Flavor	3.0	3.0
Powdered Cellulose	3.0	3.0
Egg Product	3.0	3.0
Added Amino Acids^d^	2.5	2.3
Soybean Oil	1.0	1.0
Calcium Sulfate	1.2	1.0
Calcium Carbonate	0.9	0.5
Mineral Premixes^e^	0.4	0.4
Vitamin Premixes^f^	0.6	0.6
Taurine	0.2	0.2

^a^Values are shown as percent of the total recipe. Test foods were formulated for cats with renal disease. Ingredients included at < 2.0% in test recipes (not shown) included lactic acid, potassium citrate, choline chloride, and natural flavors.

^b^HIGH-Ca:P provides 2.4 g/Mcal Ca and 1.3 g/Mcal P with a Ca:P ratio of 1.8:1.

^c^MOD-Ca:P provides 1.8 g/Mcal Ca and 1.3 g/Mcal P with a Ca:P ratio of 1.4:1.

^d^The crystalline amino acids L-Thr, L-Arg, DL-Met, L-Cys, L-Trp, and/or L-Lys were added to diets as needed to balance amino acid content across test foods.

^e^Mineral premixes included calcium carbonate as a carrier for the blend. The individual mineral compounds included ferrous sulfate, zinc oxide, copper sulfate, manganese oxide, calcium iodate, and sodium selenite.

^f^Vitamin premixes included rice hulls as a carrier for the blend. The individual vitamin compounds included vitamin A, vitamin D, vitamin E, thiamine, riboflavin, pyridoxine, vitamin B12, ascorbic acid, niacin, pantothenic acid, folic acid, biotin, and beta-carotene.

**Table 2:** Analyzed nutrient composition of test foods on a dry matter and a caloric basis.

	Dry Matter Basis			g per 1000 kcal		
Analyte	Prefeed	HIGH-Ca:P	MOD-Ca:P	Prefeed	HIGH-Ca:P	MOD-Ca:P
Calories (ME Atwater)^a^, kcal/kg	4049	4301	4369	–	–	–
Crude Protein, %	37.6	29.6	29.4	92.9	68.8	67.2
Crude Fat, %	15.2	21.7	22.6	37.6	50.4	51.7
Crude Fiber, %	1.5	2.9	2.9	3.7	6.7	6.6
Total Dietary Fiber, %	8.6	7.5	9.5	21.2	17.4	21.8
Ash, %	4.6	5.2	4.5	11.3	12.0	10.4
Arginine, %	1.89	1.74	1.71	4.67	4.05	3.91
Histidine, %	0.84	0.60	0.60	2.07	1.39	1.37
Isoleucine, %	1.53	1.10	1.10	3.78	2.56	2.52
Leucine, %	4.60	3.45	3.44	11.35	8.02	7.87
Lysine, %	1.75	1.62	1.61	4.33	3.78	3.69
Met + Cys, %	1.75	1.44	1.32	4.33	3.35	3.03
Phe + Tyr, %	3.22	2.36	2.31	7.94	5.49	5.28
Threonine, %	1.38	1.76	1.72	3.41	4.10	3.94
Tryptophan, %	0.35	0.30	0.29	0.87	0.70	0.66
Valine, %	1.21	0.89	0.85	2.99	2.06	1.96
Taurine, %	0.37	0.25	0.25	0.92	0.57	0.56
Calcium, %	0.66	1.04	0.77	1.63	2.42	1.77
Phosphorus, %	0.70	0.58	0.57	1.73	1.34	1.30
Sodium, %	0.38	0.25	0.26	0.94	0.58	0.59
Chloride, %	0.56	0.67	0.59	1.39	1.57	1.34
Potassium, %	0.69	0.83	0.79	1.70	1.94	1.81
Magnesium, %	0.113	0.083	0.083	0.278	0.194	0.191
Sulfur, %	0.82	0.76	0.67	2.02	1.76	1.54
Vitamin D (Cholecalciferol)^b^	2208	2672	2735	3540	2353	2345
DCAD^c^, mEq/kg DM	−357	−129	−185	–	–	–

^a^ME, metabolizable energy. Calculated by modified Atwater equation.

^b^Expressed as IU/kg and IU/1000 kcal.

^c^DCAD, dietary cation anion difference, was calculated as follow using total sulfur: DCAD = (49.9 × Ca) + (82.3 × Mg) + (43.5 × Na) + (25.6 × K)−(64.6 × P)−(62.4 × S)−(28.2 × Cl) [19–21].

Blood and urine samples were collected at baseline (end of prefeed/washout) and days 28 and 56 of each treatment period. Cats were fed once daily to maintain ideal BW and food intake was recorded. Fresh water was offered *ad libitum*. Cats were weighed weekly, and body condition score was assessed monthly to adjust feeding amounts. Blood samples were collected via jugular venipuncture. Urine samples were collected via cystocentesis. At no time were animals subjected to any procedures expected to cause pain or distress.

### Analyses

Complete blood count in whole blood was determined using Sysmex XT-2000iV (Sysmex Corp., Kobe, Hyogo, Japan). Serum chemistry was determined using Roche Cobas c501 (Roche Diagnostics Corp., Indianapolis, IN). Whole blood taurine (nmol/L) was analyzed by the Amino Acid Laboratory (University of California, School of Veterinary Medicine, Davis, CA) in accordance with established methods.

Serum iCa, calcitriol, calcifediol, and PTH were analyzed by Michigan State University Veterinary Diagnostic Laboratory (Michigan State University Veterinary Diagnostic Laboratory, Lansing, MI), and serum FGF-23 by IDEXX (IDEXX Laboratories, Inc., Westbrook, ME) in accordance with established methods. Urinalysis was completed using IDEXX Veterinary UA Strips (IDEXX Laboratories, Inc., Westbrook, ME). Microscopic sediment evaluation was completed using Olympus System Microscope Model CX41 (Olympus Life Science, Waltham, MA). Urine specific gravity (USG) was determined using a hand-held veterinary total solids refractometer (temperature-compensated). Urine protein to creatinine ratio (UPC) was calculated as urine protein concentration/urine creatinine concentration. Fractional excretion of calcium was calculated as urine calcium concentration/serum calcium concentration divided by urine Cr concentration/serum Cr concentration × 100. Fractional excretion of phosphorus was calculated as urine phosphorus concentration/serum phosphorus concentration divided by urine Cr concentration/serum Cr concentration × 100. Struvite and calcium oxalate (CaOx) relative supersaturation (RSS) were calculated using EQUIL-HL21 software (Hill’s Pet Nutrition, Inc., Topeka, KS) as previously described [22].

### Statistical analyses

The normality of data distribution was tested using the UNIVARIATE procedure in SAS v.9.4 (SAS Institute, Cary, NC) on the residual of the data. In instances where data did not meet normality assumptions, statistical analysis was performed on the natural logarithm transformation of the data. Data were back transformed for reporting purposes. Data were analyzed using a linear mixed model with Diet, Day, Period, and all associated interactions included as fixed effects. Interactions with Period were not significant and were subsequently removed from the model. To account for the correlation between the repeated measurements, an appropriate variance-covariance structure was selected using the AICC fit statistic. The NOBOUND option was used to allow for negative variance component estimates. The Kenward-Roger adjustment (DDFM = KR) was used to adjust the error degrees-of-freedom in the F-test and standard error for the means for the presence of multiple random effects in the model. Day was included as a repeated measure, and Animal was included as a random effect nested within Diet. LS Means were separated using Tukey’s post-hoc adjustment. In addition, regression models were run on the correlation of urine CaOx RSS with blood iCa and tCa for all cats and for cats consuming each type of diet.

Effects were considered significant when *P* ≤ 0.050 and a statistical trend when *P* ≤ 0.100. Results are presented as mean ± SD or pooled SEM as appropriate.

## Results

### Animals

Two of the 11 cats were removed during the course of the study, both during the second Period of the crossover (both consuming HIGH-Ca:P) because of poor food intake and weight loss. Data from these two cats were excluded from statistical analysis.

Period effects were observed for BW and metabolic body weight (MBW; *P* < 0.005 and 0.007, respectively; Table 3); cats weighed more in Period 1 compared with Period 2. A Diet by Day interaction was observed for BCS (*P* = 0.041), but with Tukey’s post-hoc adjustment, there were no significant differences (*P* > 0.050). A Day effect was observed for all measures of food intake (*P* < 0.050). Intake was greater during baseline feeding than during both treatment periods when cats were receiving test foods (*P* < 0.050), and caloric intake was greater at baseline compared with day 28 of treatment (*P* = 0.016). The prefeed/washout food had a lower caloric density than either of the test foods (Table 2). No other effects were observed for BW, BCS, or food intake data (*P* > 0.050).

**Table 3:** Body weight, body condition score, and food intake of cats consuming therapeutic renal foods with varying Ca:P ratios^a^ in a 56-day crossover study.

	Day 0		Day 28		Day 56			*P-Values*			
Variable	HIGH-Ca:P	MOD-Ca:P	HIGH-Ca:P	MOD-Ca:P	HIGH-Ca:P	MOD-Ca:P	SEM	Diet	Day	Diet x Day	Period
BW, kg	5.18	5.15	5.12	5.14	5.04	5.14	0.28	0.273	0.060	0.150	0.005
MBW, kg^0.75^	3.43	3.41	3.40	3.41	3.35	3.40	0.14	0.230	0.067	0.179	0.007
BCS	3.21	3.01	2.99	3.13	2.99	3.11	0.088	0.664	0.684	0.041	0.196
Intake, g/d	57.92	55.77	49.35	50.28	50.86	51.84	2.27	0.946	< 0.001	0.441	0.071
Caloric intake, kcal/d	222.93	214.57	200.48	205.32	206.6	211.73	9.01	0.911	0.021	0.378	0.070
Intake, g/kg^0.75^/d	17.0	16.4	14.6	14.8	15.2	15.3	0.54	0.874	< 0.001	0.606	0.027
Caloric intake, kcal/kg^0.75^/d	65.4	63.2	59.2	60.5	61.7	62.6	2.1	0.998	0.044	0.541	0.026

^a^HIGH-Ca:P provides 2.4 g/Mcal Ca, 1.3 g/Mcal P, and a Ca:P ratio of 1.8:1; MOD-Ca:P provides 1.8 g/Mcal Ca, 1.3 g/Mcal P, and a Ca:P ratio of 1.4:1.

### Hormonal and regulatory pathways affecting calcium homeostasis

Although mean iCa stayed within the normal reference interval throughout the study (Table 4), Day effects were observed for both iCa and tCa (*P* = 0.003 and 0.001, respectively). There was a trend for cats fed HIGH-Ca:P food to have higher iCa,(*P* = 0.067), but not tCa, compared with cats fed MOD-Ca:P food. Regardless of dietary treatment, iCa and tCa were increased compared with baseline when cats were consuming test foods, both of which had higher calcium content than prefeed food (Table 2). No Diet by Day interactions were observed for iCa, but a period effect was observed (*P* = 0.042); iCa was higher in Period 2 compared with Period 1. No Diet, Diet by Day, or Period effects were observed for tCa. Although mean FGF-23 concentration was increased above the reference interval at baseline in cats designated to consume HIGH-Ca:P food, baseline means were similar between the two groups of cats (Table 4; *P* = 0.989). There was a trend for cats fed HIGH-Ca:P food to have higher FGF-23 compared with cats fed MOD-Ca:P food (*P* = 0.058). A Day effect was observed for FGF-23 (*P* = 0.006); FGF-23 was increased on day 56 compared with baseline (*P* = 0.005), and mean FGF-23 concentrations for both dietary treatments were above the normal reference interval on days 28 and 56 of treatment. No Diet by Day nor Period effects were observed.

**Table 4:** Mean hormonal and regulatory parameters affecting calcium homeostasis in cats consuming therapeutic renal foods with varying Ca:P ratios^a^ in a 56-day crossover study.

	Normal Reference Range	Day 0		Day 28		Day 56			*P-Values*			
Variable		HIGH-Ca:P	MOD-Ca:P	HIGH-Ca:P	MOD-Ca:P	HIGH-Ca:P	MOD-Ca:P	SEM	Diet	Day	Diet x Day	Period
Ionized calcium, mmol/L	1.10-1.40	1.269	1.246	1.368	1.336	1.360	1.285	0.038	0.067	0.003	0.575	0.042
Calcium, mg/dL	8.80-11.30	9.92	9.74	10.73	10.51	10.99	10.39	0.33	0.113	0.001	0.638	0.982
Phosphorus, mg/dL	3.70 - 6.60	4.66	4.49	4.21	4.17	4.36	4.23	0.24	0.400	0.050	0.916	0.475
Parathyroid hormone, pmol/L	0.7–3.4	3.1	2.7	1.2	3.2	1.1	2.2	0.54	0.020	0.046	0.062	0.336
Calcitriol, pmol/L	13.0–145.0	33.9	24.7	14.7	17.3	10.9	12.6	6.6	0.627	0.003	0.222	0.050
Calcifediol, nmol/L	93 - 327	124	124	116	121	110	119	13	0.094	0.011	0.407	0.975
FGF-23, pg/mL	≤ 299	466	228	1001	454	1424	834	360	0.058	0.006	0.679	0.420
Calcium Fractional Excretion, %		0.295	0.344	0.451	0.346	0.512	0.413	0.073	0.384	0.048	0.272	0.502
Phosphorus Fractional Excretion, %		15.7	16.2	4.7	10.9	8.6	12.6	2.1	0.050	0.006	0.230	0.433

^a^HIGH-Ca:P provides 2.4 g/Mcal Ca, 1.3 g/Mcal P, and a Ca:P ratio of 1.8:1; MOD-Ca:P provides 1.8 g/Mcal Ca, 1.3 g/Mcal P, and a Ca:P ratio of 1.4:1.

Serum phosphorus, calcitriol, and calcifediol concentrations were unaffected by Diet or Diet by Day interaction (Table 4; *P* > 0.050), but Day effects were observed (*P* ≤ 0.050). Serum phosphorus was lower on day 28 of treatment compared with baseline (*P* = 0.047), but otherwise was similar across study days. Calcitriol was lower on days 28 and 56 compared with baseline (*P* = 0.009 and 0.005, respectively), and calcifediol was lower on day 56 compared with baseline (*P* = 0.008). A period effect was observed for calcitriol (*P* = 0.050); calcitriol was higher in Period 1 compared with Period 2.

An extreme outlier was observed for PTH on day 28 in one cat consuming MOD-Ca food (44.8 pmol/L). This value was more than three standard deviations from the mean, and therefore, was removed from the dataset for statistical analysis. All other data for this cat was included in the statistical analysis. A Diet effect was observed for PTH (Table 4; *P* = 0.020). Although PTH concentrations were similar at baseline (*P* = 0.999) and within the normal reference interval throughout the study, mean PTH concentrations were higher when cats were fed MOD-Ca:P food compared with HIGH-Ca:P food. A Day effect was also observed for PTH (*P* = 0.046), whereby PTH concentrations were lower on day 56 compared with baseline (Table 4; *P* = 0.040). A trend was observed for the PTH Diet by Day interaction (*P* = 0.062), whereby cats fed HIGH-Ca:P food tended to have lower PTH on days 28 and 56 compared with baseline (*P* = 0.067 and 0.058, respectively). Including the outlier made the PTH Diet by Day interaction *P*-value more significant at 0.022 vs. 0.062. Inclusion or exclusion of the outlier did not alter the interpretation of the results, which is that PTH was higher when cats were fed MOD Ca:P food vs. HIGH Ca:P food.

A Day effect was observed for fractional excretion of calcium (Table 4; *P* = 0.048). Fractional excretion of calcium was increased compared with baseline on day 56 (*P* = 0.041). Neither Diet nor Diet by Day interaction were observed for fractional excretion of calcium. A Diet effect was observed for fractional excretion of phosphorus (*P* = 0.050). Although similar at baseline (*P* = 1.000), phosphorus excretion was lower when cats were fed HIGH-Ca:P food compared with MOD-Ca:P food. A Day effect was also observed for fractional excretion of phosphorus (*P* = 0.006); phosphorus excretion was decreased from baseline on day 28 of treatment (*P* = 0.004), but was similar to baseline on day 56 (*P* = 0.203).

### Azotemia biomarkers

Azotemia biomarkers, including serum SDMA, Cr, blood urea nitrogen (BUN), and the BUN:Cr ratio were unaffected by Diet or Diet by Day interaction (Table 5; *P* > 0.050). Day effects were observed for all azotemia biomarkers (*P* < 0.050). Serum Cr and SDMA were higher on days 28 and 56 compared with baseline (*P* < 0.002); BUN and BUN:Cr were higher at baseline compared with days 28 and 56 (*P* < 0.001). Period effects were observed for SDMA and BUN concentrations, and BUN:Cr ratio (*P* < 0.020); all were lower in Period 1 compared with Period 2.

**Table 5:** Mean renal function biomarkers in cats consuming therapeutic renal foods with varying Ca:P ratios^a^ in a 56-day crossover study.

	Normal Reference Range	Day 0		Day 28		Day 56			*P*-Values			
Variable		HIGH-Ca:P	MOD-Ca:P	HIGH-Ca:P	MOD-Ca:P	HIGH-Ca:P	MOD-Ca:P	SEM	Diet	Day	Diet x Day	Period
SDMA, µg/dL	< 15	11.7	11.9	14.0	13.5	13.4	13.4	0.71	0.768	< 0.001	0.703	< 0.001
Creatinine, mg/dL	0.70 - 1.80	1.3	1.31	1.52	1.52	1.52	1.53	0.109	0.668	< 0.001	0.991	0.755
BUN, mg/dL	15.0 - 31.0	25	25.9	22.4	22.2	22.2	22.6	1.43	0.561	< 0.001	0.740	0.005
BUN/Cr Ratio	12.1 - 28.6	19.6	20.2	15.3	15.0	15.1	15.0	1.08	0.909	< 0.001	0.662	0.016

^a^HIGH-Ca:P provides 2.4 g/Mcal Ca, 1.3 g/Mcal P, and a Ca:P ratio of 1.8:1; MOD-Ca:P provides 1.8 g/Mcal Ca, 1.3 g/Mcal P, and a Ca:P ratio of 1.4:1.

### Food effects on urine parameters and risk of calcium oxalate stone formation

The calculated dietary cation anion difference (DCAD) was −185 mEq/kg DM for MOD-Ca:P food and −129 mEq/kg DM for HIGH-Ca:P food (Table 2). A Diet effect was observed for urine pH (Table 6; *P* = 0.040), whereby urine pH was higher when cats were fed HIGH-Ca:P food compared with MOD-Ca:P food. Urine total protein was higher (*P* = 0.010) and UPC ratio (*P* = 0.062) tended to be higher when cats were fed HIGH-Ca:P food compared with MOD-Ca:P food. A Diet by Day interaction for urine sulfate (*P* = 0.043) was observed, but with Tukey’s post-hoc adjustment, there were no significant differences. No significant effects were observed for other urinary variables, including CaOx and struvite RSS.

**Table 6:** Mean urine parameters in cats consuming therapeutic renal foods with varying Ca:P ratios^a^ in a 56-day crossover study.

	Day 0		Day 28		Day 56			*P-Values*			
Variable	HIGH-Ca:P	MOD-Ca:P	HIGH-Ca:P	MOD-Ca:P	HIGH-Ca:P	MOD-Ca:P	SEM	Diet	Day	Diet x Day	Period
Ammonium, M	0.215	0.207	0.169	0.153	0.124	0.176	0.031	0.542	0.002	0.112	0.311
Calcium, mg/dL	58	61	130	85	155	103	29	0.074	0.004	0.335	0.853
Chloride, M	0.122	0.115	0.112	0.119	0.095	0.121	0.020	0.380	0.627	0.315	0.969
Creatinine, mg/dL	227	221	314	293	302	290	39	0.408	< 0.001	0.900	0.973
Citric acid, µmol/L	4050	3107	5563	5958	6852	5815	1577	0.433	0.004	0.640	0.968
Magnesium, ppm	164	140	116	106	105	120	17	0.436	< 0.001	0.140	0.295
Osmolality, mOsm/kg	1570	1567	1545	1543	1339	1439	166	0.544	0.010	0.622	0.466
Oxalate, µmol/L	864	832	659	695	760	787	114	0.856	0.050	0.857	0.291
pH	5.83	5.83	5.93	5.84	5.92	5.79	0.047	0.040	0.371	0.255	0.185
Phosphorus, mg/dL	1294	1281	706	967	660	1000	194	0.172	0.010	0.528	0.298
Potassium, ppm	3955	3741	4784	5065	4992	5204	525	0.636	< 0.001	0.498	0.987
Sodium, ppm	1078	1179	1014	1035	939	1030	229	0.597	0.630	0.962	0.316
Specific gravity	1.035	1.036	1.036	1.034	1.032	1.035	0.0035	0.699	0.329	0.309	0.187
Sulfate, M	0.0577	0.0601	0.065	0.0568	0.0479	0.0623	0.0091	0.442	0.408	0.043	0.895
Total protein, mg/dL	40	40	48	42	46	36	5	0.010	0.482	0.789	0.106
UPC ratio	0.32	0.20	0.18	0.16	0.19	0.13	0.052	0.062	0.029	0.489	0.066
CaOx RSS	4.74	5.69	6.69	4.96	8.80	6.58	1.23	0.147	0.009	0.106	0.112
Struvite RSS	0.416	0.293	0.047	0.061	0.048	0.091	0.082	0.374	< 0.001	0.116	0.115

a HIGH-Ca:P provides 2.4 g/Mcal Ca, 1.3 g/Mcal P, and a Ca:P ratio of 1.8:1; MOD-Ca:P provides 1.8 g/Mcal Ca, 1.3 g/Mcal P, and a Ca:P ratio of 1.4:1.

A Day effect was observed for most urine parameters (Table 6). Compared with baseline, calcium tended to be higher on day 28 and was higher on day 56 (*P* = 0.052 and 0.003, respectively). The CaOx RSS on day 56 was increased compared with baseline and tended to be higher than CaOx RSS on day 28 (*P* = 0.009 and 0.063, respectively). Serum iCa was significantly (*P* < 0.001) correlated to CaOx RSS (for all cats R^2^ = 0.321; for cats consuming High-Ca:P food R^2^ = 0.563). Serum tCa was also significantly (*P* < 0.001) correlated to CaOx RSS (for all cats R^2^ = 0.408; for cats consuming High-Ca:P food R^2^ = 0.656).

All other CBC and serum chemistries findings are reported in S1 Table.

## Discussion

Because phosphorus restriction is a primary goal in CKD management to prevent hyperphosphatemia and secondary renal hyperparathyroidism [4,5,10,11], we and others have investigated feeding foods with varying phosphorus contents and Ca:P ratios for healthy colony cats and cats with CKD [12,13,15,17,23–25]. Based on long-term feeding studies, it has been suggested that a food containing 1 g/Mcal of inorganic phosphorus results in estimated tubular phosphate concentration <2.5 mmol/L and can be fed without detectable adverse effects in the kidney tubules [26]. The AAFCO adult cat maintenance minimum phosphorus recommendations is 1.25 g/Mcal and 1.5 g/Mcal for calcium [27]. It is also recommended to maintain the Ca:P ratio above 1.0:1 to avoid nutritional secondary hyperparathyroidism and the adverse effects of phosphorus consumption on kidney function in cats [23,28].

In this study, cats with IRIS Stage 1 and 2 CKD maintained serum iCa and tCa concentrations within biological reference intervals when fed either MOD-Ca:P food (1.8 g/Mcal Ca, 1.3 g/Mcal P, Ca:P 1.4:1) or HIGH-Ca:P food (2.4 g/Mcal Ca, 1.3 g/Mcal P, Ca:P 1.8:1) for 56 days. Serum iCa and tCa concentrations increased after consuming both test foods compared with prefeed/washout food, most likely because calcium content in both test foods was higher than in the prefeed food (1.6 g/Mcal Ca, 1.7 g/Mcal P, Ca:P ratio of 0.9:1). A Period effect for iCa reflected higher iCa the longer cats were on the study. It is unclear from these results whether increasing calcium concentrations reflect disease progression or response to the test foods. The small sample size (n = 9) limits statistical power and we cautiously reported a statistical trend for cats fed HIGH-Ca:P to have higher iCa compared with cats fed MOD-Ca:P food, implying that a potential effect may exist that might have been detected with a larger sample size, rather than a true absence of dietary impact.

In a recent case series, researchers found ionized hypercalcemia in cats with CKD (*n* = 3) or idiopathic hypercalcemia (*n* = 7) that were fed foods with a Ca:P ratio >1.4:1, Ca > 2 g/Mcal, or both [25]. We have also shown in a randomized, controlled clinical trial that feeding a moderately phosphate-restricted food (1.8 g/Mcal Ca, 1.5 g/Mcal P, Ca:P 1.2:1) for 140 days to cats with early stage CKD maintained iCa concentrations within the normal reference range, whereas feeding a more highly phosphate restricted food, but also a more Ca enriched food (2.3 g/Mcal Ca, 1.1 g/Mcal P, Ca:P 2.0:1) resulted in higher serum iCa and tCa concentrations (hypercalcemic range) after 28 days [17].

In a longitudinal case study, researchers showed that feeding a low P (1.62 g/Mcal Ca, 0.84 g/Mcal P, Ca:P 1.9:1) food for 18 months, to cats with IRIS stages 1–2 diet-induced CKD, was associated with hypercalcemia [15]. Increasing dietary phosphorus content and reducing the Ca:P ratio (2.2 g/Mcal Ca, 1.4 g/Mcal P, Ca:P 1.4–1.6:1) and feeding for 22 months resulted in tCa returning to the reference range [15]. Then in a retrospective study, researchers observed that feeding a moderately phosphate-restricted food (2.0 g/Mcal Ca, 1.5 g/Mcal P, Ca:P 1.3:1) resulted in normalization of iCa in client-owned cats that developed hypercalcemia while eating a highly phosphate-restricted renal food (1.5 g/Mcal Ca, 0.8 g/Mcal P, Ca:P 1.9:1) [13]. In another retrospective study, researchers observed that feeding a phosphate-restricted food (0.7–1.1 g/Mcal P, Ca:P 1.3–1.9:1) may lead to hypercalcemia in some cats with stage 2–3 azotemic CKD [12]. Feeding a moderately phosphate-restricted food (1.9 g/Mcal Ca, 1.6 g/Mcal P, Ca:P 1.2:1) for 18 months to healthy older client-owned cats was well tolerated [24].

The source of P in the foods (organic vs inorganic) was the same for both test foods in this study, so P source was not addressed as a contributor to hypercalcemia [29,30]. Our goal in this study was to hold absolute P content and bioavailability constant while changing the Ca:P ratio. In a recent study with healthy adult cats, six experimental foods were fed to determine the effect of three dietary Ca:P ratios (approximately 1.0:1, 1.5:1, and 2.0:1) in foods supplemented with either a high (1.5 g/Mcal P) or low (0.75 g P/Mcal) amount of highly-soluble, inorganic sodium tripolyphosphate on postprandial plasma P, iCa and PTH [31]. They found that increasing the Ca:P ratio to >1.6 and reducing the level of added soluble P in the food, mitigated the postprandial rise in plasma P and PTH concentrations [31]. In that study, all foods decreased 1–6 hour postprandial blood iCa concentrations from baseline [31].

The development of hypercalcemia in cats with early stage CKD may also be related to decreasing GFR. In this study, cats showed progression of CKD with increases in SDMA and Cr and a decrease in BUN:Cr ratio. Increased renal tubular reabsorption of calcium seems less likely as a cause of increasing iCa and tCa concentrations as fractional excretion of calcium increased from baseline in cats fed both HIGH-Ca:P and MOD-Ca:P foods. As both test foods had higher Ca content than the prefeed/washout food, we suspect that increased calcium intestinal absorption underlies increasing iCa and tCa concentrations, although we cannot rule out increased bone resorption as a contributor to increasing iCa and tCa concentrations in these CKD cats [14]. Both test foods had similar, but almost double crude fiber content compared with prefeed food, which may have helped to decrease intestinal absorption of calcium when feeding the higher-Ca test foods [32].

PTH concentrations were decreased in cats consuming HIGH-Ca:P compared with cats consuming MOD-Ca:P, but remained within the reference interval. Dietary phosphate restriction has been shown to reduce PTH concentrations in cats with CKD [10,17], although both test food had similar phosphate content in this study. The tendency for iCa to increase after consuming HIGH-Ca:P food may have played a role in decreasing PTH concentrations as increased iCa was associated with lower PTH concentrations in client-owned cats with azotemic CKD [14].

Cats fed both test foods had increased FGF-23 concentrations above baseline and above the reference interval, although cats fed HIGH-Ca:P food tended to have higher FGF-23 concentrations compared with cats consuming MOD-Ca:P food. Increased FGF-23 concentrations are an early pathological finding in CKD in humans and in cats [33–37]. FGF-23 is a phosphatonin that is important in the control of phosphate metabolism [3,33,35]. It is produced by osteocytes and osteoblasts in bone in response to hyperphosphatemia and increased calcitriol. In renal tubules, FGF-23 binds to FGF receptors along with the cofactor Klotho and inhibits expression of sodium-dependent phosphate cotransporters. This decreases phosphorus reabsorption in order to maintain phosphorus balance. The presence of Klotho is required to allow FGF-23 to exert its action in the kidney. Klotho plasma levels and Klotho urinary excretion decrease with progressive CKD [38,39]. In our study, fractional excretion of phosphorus decreased from baseline in cats fed both HIGH-Ca:P and MOD-Ca:P foods. However, fractional excretion of phosphorus decreased to a greater extent in cats fed HIGH-Ca:P compared with MOD-Ca:P food. Higher urine total protein after feeding HIGH-Ca:P food compared with MOD-Ca:P food (also noted in the UPC ratio), is consistent with deterioration of renal podocyte health [40].

FGF-23 also regulates calcifediol metabolism by inhibiting expression of the 1α-hydroxylase enzyme, leading to less formation of calcitriol. Simultaneously, FGF-23 increases activity of the 24-hydroxylase enzyme, the major enzyme that catabolizes calcifediol and calcitriol. In our study, serum calcitriol decreased after feeding both HIGH-Ca:P and MOD-Ca:P foods, consistent with the consequences of increased FGF-23 concentrations.

Total calcium has also been shown to be an independent predictor of FGF-23 in cats with CKD [3]. The role of calcium in the regulation of FGF-23 remains incompletely characterized, but studies in genetically modified mice indicate that increasing serum calcium increases FGF-23 [41–43]. In one study, the best correlation between calcium, phosphorus and serum FGF-23 was found between FGF-23 and the calcium × phosphorus (Ca × P) product [41]. This suggests that the regulation of FGF-23 by both calcium and phosphorus is fundamentally important to ensure that the Ca × P product remains within the physiological range. In our study, the Ca × P product was higher for cats fed the HIGH-Ca:P food (baseline to 56 days, means, 3.72 to 3.86 mmol^2^/L^2^) compared with cats fed the MOD-Ca:P food (baseline to 56 days, means, 3.53 to 3.55 mmol^2^/L^2^). Cats with an increased Ca × P product >5.6 mmol^2^/L^2^ are at risk of soft tissue mineralization [44].

Nephrocalcinosis has been found in 61% of CKD cats, and in 81% of CKD cats with hypercalcemia [45]. Urine stone formation risk metrics showed that cats in this study fed both test foods had higher CaOx RSS compared with prefeed food, but all values were within the metastable range for CaOx (<12) [46]. Fractional excretion of calcium was also increased from baseline in cats fed both HIGH-Ca:P and MOD-Ca:P foods. Increased urine calcium concentrations were primarily responsible for the higher CaOx RSS [46]. The highly significant correlation between CaOx RSS in the urine, and both iCa and tCa in the blood for cats consuming High Ca:P food suggests that food influenced that change. Thus, cats consuming test foods with higher calcium content than prefeed food had higher serum calcium concentration, higher calcium fractional excretion, and were at greater risk of CaOx crystal formation as determined by the CaOx RSS test.

Oxalate concentrations were lower after consuming both test foods, which would theoretically lower risk of CaOx crystal formation. The urine concentrations of citric acid and potassium were higher in cats fed test foods compared with baseline prefeed food throughout the feeding study. Both test foods contained added potassium citrate, and analyzed potassium content in test foods was higher compared with baseline prefeed food. Higher citrate and potassium concentrations in urine would also counteract the increased CaOx RSS in cats fed test foods [46].

Feeding a magnesium-enriched, phosphate-restricted diet stabilizes serum calcium and FGF-23 concentrations in cats with normal or low serum magnesium levels [43]. Cats with CKD fed such a diet showed greater stability in ionized calcium and FGF-23 compared with cats fed phosphate-restricted diets alone [43]. Serum total magnesium was not measured in our study.

Limitations of this study include the small number of cats participating and the short-term of the study period. In addition, cats were IRIS Stage 1or 2 CKD, and compensatory mechanisms often keep iCa, FGF-23, and PTH within normal ranges in early stage CKD. A longitudinal follow-up study could help capture slower or cumulative effects of dietary Ca:P ratios. Future studies should include more cats, a longer feeding period, additional foods with a broader range of Ca:P ratios, and a renal therapeutic prefeed food.

In conclusion, in this small, crossover study (56 days duration for each food), cats with early-stage but progressive CKD maintained iCa and tCa within the reference interval when fed foods with Ca:P ratios of 1.4:1 or 1.8:1. Cats consuming the High-Ca:P food had lower PTH concentrations, reduced fractional excretion of phosphorus, higher urine total protein concentration, and a statistical trend for higher iCa and FGF-23 concentrations. This study provides preliminary evidence regarding the role of Ca:P ratio in feline CKD foods. Additional studies are needed with a broader range of Ca:P ratios to make definitive conclusions.

## Supporting information

S1 Table Additional summary statistics for CBC and serum chemistry parameters for cats consuming therapeutic renal foods with varying Ca:P ratios in a 56-day crossover study. (XLSX)

## Abbreviations

AAFCO: Association of American Feed Control Officials
BW: body weight
BUN: blood urea nitrogen
CaOx: calcium oxalate
Ca:P: calcium-to-phosphorus ratio
CKD: chronic kidney disease
Cr: creatinine
DCAD: dietary cation anion difference
FGF-23: fibroblast growth factor 23
GFR: glomerular filtration rate
iCa: ionized serum calcium
IRIS: International Renal Interest Society
HIGH-Ca:P: high level of calcium food with 2.4 g/Mcal Ca and 1.3 g/Mcal P and a Ca:P ratio of 1.8:1
MBW: metabolic body weight
MOD-Ca:P: moderate level of calcium food with 1.8 g/Mcal Ca and 1.3 g/Mcal P and a Ca:P ratio of 1.4:1
PTH: parathyroid hormone
RSS: relative super saturation
SDMA: symmetric dimethylarginine
tCa: total serum calcium
UPC: urine protein to creatinine ratio
USG: urine specific gravity
